# Nanoparticle System Based on Amino-Dextran as a Drug Delivery Vehicle: Immune-Stimulatory CpG-Oligonucleotide Loading and Delivery

**DOI:** 10.3390/pharmaceutics12121150

**Published:** 2020-11-27

**Authors:** Hien V. Nguyen, Katrin Campbell, Gavin F. Painter, Sarah L. Young, Greg F. Walker

**Affiliations:** 1 School of Pharmacy, University of Otago, Dunedin 9016, New Zealand; hien.nguyen@postgrad.otago.ac.nz; 2 Department of Pathology, University of Otago, Dunedin 9016, New Zealand; katrin.campbell@otago.ac.nz; 3 The Ferrier Research Institute, Victoria University of Wellington, Wellington 5040, New Zealand; gavin.painter@vuw.ac.nz; 4 School of Medical Sciences, Faculty of Medicine and Health, University of Sydney, Sydney 2006, NSW, Australia; sarah.l.young@sydney.edu.au

**Keywords:** dextran nanoparticle, desolvation, vaccine formulation, CpG oligonucleotide, CpG adsorption, CpG conjugation

## Abstract

The aim of this study is to prepare and characterize an amino-dextran nanoparticle (aDNP) platform and investigate two loading strategies for unmethylated cytosine-phosphate-guanine (CpG) oligonucleotide. aDNP was prepared by desolvation of amino-dextran followed by the chemical crosslinking of amino groups. Size, surface charge, and surface morphology of aDNP was determined by dynamic light scattering and transmission electron microscopy. CpG was either loaded onto aDNP by adsorption (CpG-adsorbed-aDNP) or conjugated to aDNP (CpG-conjugated-aDNP). In vitro cytokine production by bone marrow-derived dendritic cells (BMDCs) was measured by flow cytometry. aDNPs size and zeta potential could be controlled to produce uniform particles in the size range of 50 to 300 nm, surface charge of −16.5 to +14 mV, and were spherical in shape. Formulation control parameters investigated included the anti-solvent, water-to-anti-solvent ratio, level of amine functionality of dextran, and the molar ratio of glutaraldehyde to amine. aDNP could be lyophilized without additional cryoprotectant. Unloaded cationic aDNP (+13 mV) showed acceptable in vitro hemolysis. Unloaded and CpG-loaded aDNPs showed no cytotoxicity on BMDCs. CpG-loaded nanoparticles stimulated cytokine production by BMDCs, the level of cytokine production was higher for CpG-conjugated-aDNP compared to CpG-absorbed-aDNP. aDNP is a promising new drug delivery platform as its offers versatility in loading and tuning of particle properties.

## 1. Introduction

Polymeric nanoparticles are of interest from both academic and translational perspectives as they are used in a large variety of applications, including carriers for drug delivery [1]. To date, only a few polymeric nanoparticles have been approved for clinical use or under clinical trial, such as poly lactic-co-glycolic acid (PLGA) and poly-lactic acid (PLA) nanoparticles [2]. The limitation of polymeric nanoparticles in their clinical translation potential is mainly due to the lack of biocompatibility and challenges in large-scale production. Therefore, there is an ongoing need to identify new versatile carrier materials for the fabrication of nanoparticles that are easily functionalized, biocompatible and stable [3].

Dextran is a promising material for a drug delivery carrier as it has a proven safety in human clinical applications such as plasma volume expansion and plasma substitution [4,5]. For the fabrication of dextran into a nanoparticle, dextran has been chemically modified to enable nanoparticle formation. For example, the hydroxyl groups on dextran can be esterified with lipoic acid to become an amphiphile for nanoparticle self-assembly [6]; or can be modified to form acetal moieties via dehydration reactions to render it hydrophobic for emulsion techniques [7]. Dextran has also been used to be an effective shielding agent on the surface of nanoparticles for minimizing the opsonization of nanoparticles [8].

Alternatively, the hydroxyl groups on dextran can be modified to amino functional groups that offer alternative reactivity for subsequent conjugation and/or crosslinking reactions to make molecular scaffolds. The incorporation of amino groups on the dextran polymer will enable the subsequent chemical crosslinking of polymer chains in a nanoparticle formed by the process of desolvation. The desolvation technique for making nanoparticles involves adding the polymer dissolved in an aqueous medium to an anti-solvent agent under mixing [9]. The anti-solvent, such as ethanol or acetone, decreases the hydration level and the solubility of the water-soluble polymer, leading to the nanoparticle formation. The nanoparticle is then stabilized by the addition of an amine-reactive crosslinking agent such as glutaraldehyde. This desolvation process is commonly used to prepare a nanoparticle from proteins such as gelatin and human serum albumin [10,11].

For tuning the overall properties of the particle to fit a targeted application, the non-crosslinked amino groups at the nanoparticle surface could be used for the subsequent conjugation of molecules. For example, the nanoparticle surface can be conjugated with a shielding polymer and/or cell targeting ligands for improved targeted drug delivery applications [12]. Alternatively, the surface amino group can be conjugated to a bioactive molecule with the aim of improving its biodistribution and overall efficacy and/or safety. The amino groups at the nanoparticle surface will also generate a particle with a net positive surface charge. Cationic nanoparticles have recently emerged as promising carrier systems for the effective delivery due to their cellular interaction and uptake properties, which results from their net positive surface charge [13].

An amino-functionalized nanoparticle can also be loaded with a bioactive molecule either by the covalent conjugation of the molecule to a surface amine group, or by the process of adsorption whereby in solution an anionic molecule is adsorbed to the surface of the cationic particle by electrostatic attractive forces. Unmethylated cytosine-phosphate-guanine (CpG) oligonucleotides are one class of anionic molecules that have been loaded onto the surface nanoparticles either by conjugation or adsorption method [14,15]. CpG oligonucleotides have recently gained interest in the field of drug delivery as they have been approved for use in humans as immune-stimulatory agents for vaccines for the treatment of cancer and infectious diseases [16]. To generate an immune response, CpG must traffic to the endosome of an antigen-presenting cell where it then binds to the pattern recognition receptor Toll-like receptor 9 (TLR-9) [17]. Several nanocarrier systems have been shown to increase the immune-stimulatory activity of CpG compared to the unformulated free CpG, which has been reviewed by Mutwiri et al. [18]. These nanocarriers have been loaded with CpG by using different methods including encapsulation or immobilization to the carrier surface either by adsorption or conjugation [14,15,19]. To the best of our knowledge, there have been no reports on the impact of the method of CpG immobilization to the particle surface on immune-stimulation.

This study demonstrates for the first time the fabrication of amino-functionalized dextran nanoparticles from amino-dextran by the desolvation method. Dynamic light scattering analysis shows that particle size and zeta potential of the nanoparticles can be tuned by altering processing factors and the level of amino functionalization of the dextran used. The amino-dextran nanoparticle (aDNP) system described in this work offers a versatile platform for the loading of molecules to the nanoparticle surface. This is demonstrated by using CpG that was immobilized to the nanoparticle either by conjugation or adsorption technique, followed by evaluating the in vitro immunostimulatory activity of the CpG-loaded aDNPs. Amino-dextran is a promising carrier system that offers with a wide range of modification and application possibilities.

## 2. Materials and Methods

### 2.1. Materials

Amino-dextran with the molecular weight of 70 kDa modified with either 10, 25 or 50 amino groups per polymer was purchased from Fina Biosolutions (Rockville, MD), the chemical structure is shown in Supplementary Figure S1. CpG 1668 oligonucleotide (5′-TCCATGACGTTCCTGATGCT-3′) with a phosphorothioate backbone and modified with a 3′ amine (CpG-NH_2_) was obtained from Integrated DNA Technologies (Coralville, IA, USA). Glutaraldehyde solution 25% (*w/v*) in water was purchased from Sigma-Aldrich (St. Louis, MO, USA). Quant-iT^®^ OliGreen^®^ ssDNA assay kit, cell culture media (complete Iscove’s Modified Dulbecco’s Medium (cIMDM) containing IMDM and GlutaMAX), and heat-inactivated fetal bovine serum (FBS) were purchased from Thermo Fisher Scientific (Pittsburg, PA, USA). Granulocyte-macrophage colony-stimulating factor (mGM-CSF) was obtained from BioLegend (San Diego, CA, USA). The CellTiter 96^®^ Aqueous One-solution cell proliferation assay kit (MTS) was obtained from Promega (Madison, WI, USA). Succinimidyl-6-hydrazino-nicotinamide (HyNic) and succinimidyl 4-formyl benzoate (4FB) were purchased from TriLink Biotechnologies (San Diego, CA, USA). All other chemicals were of analytical grade. Water was deionized and purified by passing through a Milli-Q water Millipore Purification System™ (Billerica, MA, USA).

### 2.2. Preparation of Amino-Dextran Nanoparticles

Amino-dextran (10 mg) was dissolved in 1 mL of deionized water and filtered through a 0.22 µm syringe filter (Millipore Corp., Billerica, MA, USA). The amino-dextran solution was then added to the anti-solvent under magnetic stirring (500 rpm) using a syringe pump (200 syringe pump; Chemyx Stafford, TX, USA) with a flow rate of 1 mL/min. Glutaraldehyde (25% *w*/*v*, 2.5 M) was mixed in the anti-solvent to give a concentration of either 90, 180, or 360 mM, this cross-linking solution (0.1 mL) was then added dropwise to the dextran suspension under magnetic stirring (500 rpm). The suspension was stirred at 500 rpm for 6 h and then dialyzed against deionized water (2 L) with three medium exchanges over 24 h at room temperature. The dialyzed nanoparticle suspension was either stored at 4 ℃ for subsequent loading experiments or freeze-dried.

### 2.3. Dynamic Light Scattering

The particle size, polydispersity index (PDI), and zeta potential of aDNP were measured using the dynamic light scattering (DLS) system (Malvern ZEN3600 Zetasizer Nano, Malvern Panalytical, Worcestershire, UK) equipped with a 633 nm laser and 173° detection optics. The sample was diluted with deionized water for size measurements, or was diluted with 10 mM NaCl for the zeta potential measurement.

### 2.4. Transmission Electron Microscopy

Nanoparticle suspension (10 µL) was added to a carbon-coated grid and was incubated for 1 min at room temperature. Excess sample was removed with a filter paper and 10 μL of 1% phosphotungstic acid in PBS buffer (pH 6.8) was added and then immediately removed. The sample was observed on a Phillips CM100 BioTWIN TEM (Philips Electron Optics, Eindhoven, The Netherlands). Images were captured using iTEM Universal Imaging Platform (SoftImaging System, Munster, Germany).

### 2.5. Freeze Drying

The nanoparticle suspension (2 mL in deionized water, 2 mg nanoparticle based on dry mass) was transferred to a 5 mL freeze-drying glass vial and was frozen on a shelf set at −40 °C for 24 h (FreeZone^®^ Triad Freeze Dry System Models 740,000 Series, Labconco, Kansas City, MO, USA). A vacuum of 0.008 mBar was then applied, the shelf temperature was maintained at −40 °C for 48 h, followed by increasing the shelf temperature to −5 °C and held for 3 h and then increasing to 20 °C for 2 h.

### 2.6. CpG Adsorption onto Amino-Dextran Nanoparticles

aDNP (0.8 mg/mL based on dry mass, 1 mL) suspended in deionized water was mixed with either 4, 8, 16, 32, or 48 µL of CpG at a concentration of 5 µg/µL in deionized water followed by a gentle shaking (100 rpm) at room temperature for 4 h. The unbound CpG was then washed with PBS buffer (pH 7.4) by five cycles of centrifugal filtration (Vivaspin 500, MWCO 10 kDa, Sartorius, Göttingen, Germany) at 12,000× *g*, 4 °C. The washed nanoparticle suspension was then quantified for CpG using the Quant-iT OliGreen^®^ ssDNA kit (Thermo Fisher Scientific).

### 2.7. Conjugation of CpG to Amino-Dextran Nanoparticles

CpG modified with an amine group on 3′-end (0.75 nmol/µL) was reacted with 4FB (162 nmol/µL in DMSO) at a molar ratio of 1:20 in the modification buffer (0.1 M NaPO4, 0.15 M NaCl, pH 8.0) for 4 h at room temperature. After the reaction was complete, the unreacted linker was removed from 4FB-modified CpG by five cycles of centrifugal filtration using a spin filter (Vivaspin 500, MWCO 3 kDa, Satorius) and was resuspended in reaction buffer (0.1 M NaPO4, 0.15 M NaCl, pH 6.0).

aDNP (4 mg/mL, 400 µg aDNP dry mass) was reacted with a 40-fold molar excess of the HyNic linker (68.9 nmol/µL in DMSO) in the modification buffer for 3 h at room temperature. The unreacted linker was removed from HyNic-modified aDNP by five cycles of centrifugal filtration using a spin filter (Vivaspin 500, MWCO 3 kDa, Satorius) and was resuspended into the reaction buffer.

The HyNic-modified aDNP was reacted with 4FB-modified CpG at a molar ratio of 1:5 for 3.5 h at room temperature. The CpG-conjugated aDNP was washed in PBS buffer (pH 7.4) by 5 cycles of centrifugal filtration (Vivaspin 500, MWCO 10 kDa). The concentration of conjugated CpG was quantified using the Quant-iT OliGreen^®^ ssDNA kit (Thermo Fisher Scientific).

### 2.8. Animals: Source and Ethics

Female C57BL/6 mice (6–12 week old) were supplied by the Biomedical Research Facility, University of Otago, Dunedin, New Zealand. Animal experiments were performed according to ethical permits provided by the University of Otago Animal Ethics Committee (DET17/17, AUP-20-55). All mice were euthanized by cervical dislocation.

### 2.9. Generation of Bone Marrow-Derived Dendritic Cells

Bone marrow-derived dendritic cells (BMDCs) were prepared from C57BL/6 mice, as previously described by Inaba et al. [20]. Femurs and tibiae from euthanized mice were isolated. The bone marrow was flushed out of the bones using PBS buffer containing 5% FBS, followed by lysing of red blood cells with ammonium chloride. The remaining cells were cultured for 7 days in cIMDM supplemented with 5% FBS and 20 ng/mL mGM-CSF.

### 2.10. In Vitro Cell Viability

The cytotoxicity against BMDCs was determined using the MTS assay kit (Promega) according to the kit manufacturer’s instructions. BMDCs suspension (100 μL) at the concentration of 50,000 cells/mL was incubated with 17.5 μL of the unloaded aDNP at the concentration of either 50, 100, 150 or 200 μg/mL (based on nanoparticle dry mass), or the CpG-loaded aDNP at nanoparticle concentration of 200 μg/mL in PBS buffer (pH 7.4) for 48 h at 37 °C and 5% CO_2_ in a flat-bottomed 96-well plate (Greiner Bio-One, Monroe, NC, USA). The MTS working solution (20 μL) was added to the well and the plate was further incubated for 4 h at 37 °C and 5% CO_2_. The absorbance was measured at a wavelength of 490 nm using a microplate reader (POLARStar Omega, BMG Labtech GmbH, Ortenberg, Germany). PBS buffer (pH 7.4) and 0.5% (*v/v*) Triton X-100 were used as negative and positive controls, respectively.

### 2.11. Hemolysis Assay

The hemolytic activity of the unloaded aDNP suspended in PBS buffer (1 mg/mL dry weight equivalent) was determined according to the method described by Jumaa et al. [21]. Blood (3 mL) from a Sprague Dawley rat was collected in a heparinized-tube and was centrifuged at 2000× *g* for 5 min. The pellet was washed three times with the isotonic saline solution (0.9% (*w*/*v*) NaCl) by centrifugation at 2000× *g* for 5 min. The washed pellet was then subsequently diluted in 11 mL of PBS buffer. A 100 µL of the erythrocyte dispersion was added to 1 mL of the aDNP suspension (equivalent to 1 mg nanoparticle dry mass), then incubated at 37 °C for 1 h with shaking at 100 rpm. The sample was then centrifuged at 2000× *g* for 15 min, the supernatant was diluted four-fold in PBS buffer and the absorbance was measured at 415 nm using a UV-Vis spectrophotometer (Ultrospec 2000, Biotech Pharmacia, Uppsala, Sweden).

### 2.12. In Vitro Cytokine Production by Bone Marrow-Derived Dendritic Cells

BMDCs (200 μL) at the concentration of 1 × 10^6^ cells/mL were incubated with CpG either in the free solution form or loaded onto the nanoparticle at a concentration of 0.4 µM in a round-bottomed 96-well plate (Greiner Bio-One, Monroe, NC, USA) for 24 h at 37 °C. The plate was then centrifuged at 350× *g*, 4 °C for 3 min and the cell supernatant was collected. Measurement of pro-inflammatory cytokines, tumor necrosis factor-α (TNF-α), interleukin-6 (IL-6), and interleukin-12p70 (IL-12p70), produced in the culture supernatants was undertaken using the bead-based LEGENDplex^TM^ kit (BioLegend) according to the protocol recommended by the kit manufacturer.

## 3. Results

### 3.1. Preparation and Characterization of Nanoparticles

aDNP was prepared from amino-dextran by the process of desolvation. This technique involves preparing amino-dextran in deionized water and then adding slowly to an anti-solvent, followed by glutaraldehyde cross-linking to stabilize the formed nanoparticles and then solvent removal by dialysis. Previous studies using the desolvation method for nanoparticle production have shown that the properties of nanoparticle can be controlled by adjusting the production parameters such as the anti-solvent, solvent to anti-solvent volume ratio, polymer concentration, and the crosslinker concentration [9,11].

**Figure 1:**
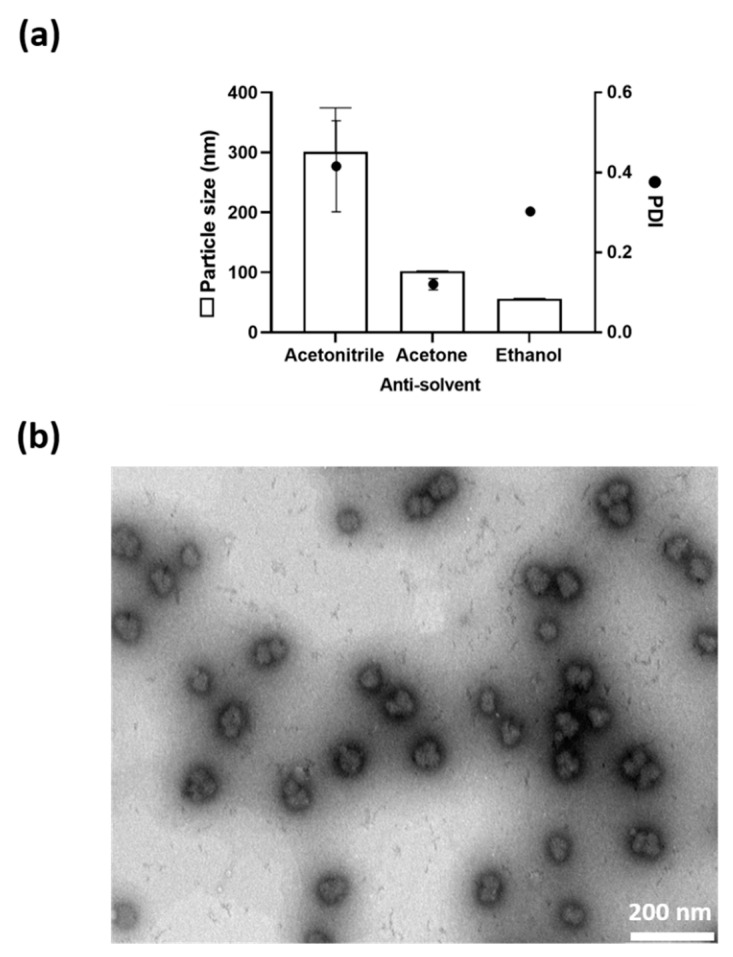
Physical characterization of the amino-dextran nanoparticles. (**a**) Effect of the anti-solvent on the particle size and the PDI of aDNP. (**b**) Transmission electron microscopy photomicrograph of aDNP produced using acetone (×93,000). Scale bar, 200 nm. aDNP: dextran with 50 amines per polymer; water-to-antisolvent volume ratio of 1:5; molar ratio of glutaraldehyde to amine of 2.5.

To understand the impact of the anti-solvent on the nanoparticle properties, aDNP was prepared using either acetonitrile, acetone, or ethanol as the anti-solvent. Analysis of the particle suspension by DLS showed that the anti-solvent influenced the particle size, while having no impact on the zeta potential (+11 to +13 mV). In particular, acetonitrile generated nanoparticles with the largest particle size of 300 nm, followed by acetone (100 nm) and then ethanol (55 nm) (Figure 1a). This decrease in particle size correlated with the increasing water-miscibility of the anti-solvent. A TEM image of the aDNP suspension prepared with acetone showed particles with a spherical shape with a size range from 75–120 nm, which was in agreement with DLS data (Figure 1b).

**Figure 2:**
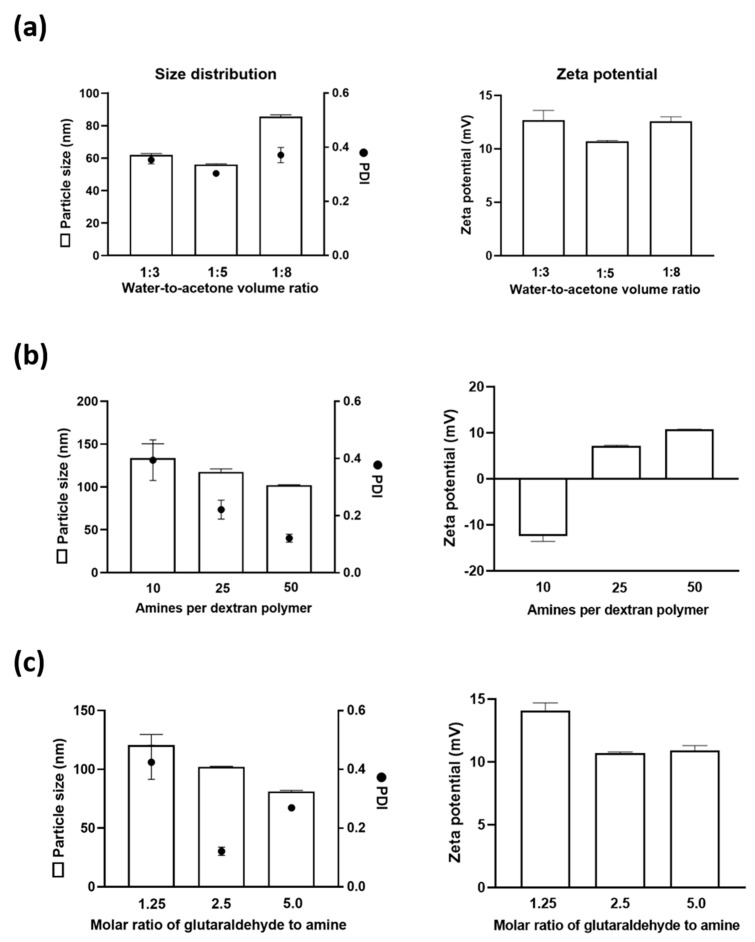
Effect of formulation parameters on the particle size, polydispersity index and surface charge of the amino-dextran nanoparticle. (**a**) Effect of water-to-antisolvent ratio: dextran with 50 amines per polymer; molar ratio of glutaraldehyde to amine (GAR) of 2.5. (**b**) Effect of amine functionality: water-to-antisolvent ratio of 1:5; GAR of 2.5. (**c**) Effect of glutaraldehyde content: dextran with 50 amines per polymer; water-to-nonsolvent ratio of 1:5. Data are presented as the mean ± S.D. (*n* = 3).

For the subsequent characterization studies, acetone was selected as the anti-solvent for the desolvation of amino-dextran. The influence of the water-to-acetone volume ratio was investigated at ratios of 1:3, 1:5, and 1:8, as presented in Figure 2a. At the solvent ratio of 1:3 and 1:5, the particle sizes were similar at around 100 nm; however, the PDI decreased from 0.46 to 0.12, respectively. Increasing the solvent ratio to 1:8, the particle size increased to 120 nm with a similar PDI of 0.08.

Amino-dextran is available with different levels of amino functionality which may impact on the cross-linking reaction, size and surface charge of the particle. aDNPs were made using acetone as the anti-solvent with a water-to-acetone ratio of 1:5. Dextran (70 kDa) modified with either 10, 25 or 50 amino groups per polymer was used to produce nanoparticles. Figure 2b shows that nanoparticles made from dextran with increasing amine functionality resulted in a decrease in particle size and PDI. The zeta potential of the nanoparticles made from dextran with 25 or 50 amino groups per polymer generated a net positive charge of +7 and +12.5 mV, respectively. This net positive surface charge is due to amine groups that were not cross-linked by glutaraldehyde. However, the nanoparticles made from dextran containing 10 amino groups per polymer had a net negative charge of −12.5 mV. This indicated that all of the amine groups at the nanoparticle surface were cross-linked by glutaraldehyde, and the carbonyl functional group introduced to the dextran resulted in a net negative charge (Supplementary Figure S1). Glutaraldehyde was used as an amine-reactive crosslinker to stabilize the amino-nanoparticle following desolvation. Previous studies have shown that the amount of glutaraldehyde impacted on both particle size and zeta potential of the crosslinked nanoparticles [22]. The molar ratio of glutaraldehyde-to-amine (GAR) used in these studies to investigate impact of other parameters was fixed at 2.5. Using amino-dextran with 50 amino groups per polymer, the impact of GAR on the particle size and surface charge was investigated at the GAR of 1.25, 2.5 and 5. As shown in Figure 2c, increasing the GAR from 1.25 to 2.5 resulted in a decrease in particle size from 120 to 100 nm and the PDI decreased from 0.42 to 0.12. Further increasing the GAR from 2.5 to 5 decreased the particle size to 80 nm, while the PDI increased from 0.12 to 0.27. With increasing the GAR from 1.25 to 2.5, the zeta potential of aDNP reduced from +14.1 to +10.7 mV, while remained unchanged with further increase of GAR to 5.

Subsequent characterisation studies evaluated aDNPs made from dextran with 50 amino groups per polymer, desolvated in acetone at a water-to-acetone ratio of 1:5, and crosslinked by glutaraldehyde at GAR of 2.5. Using these parameters, the aDNP had a particle size of 100 nm, PDI of 0.12, and a net positive charge of +12.5 mV.

### 3.2. Stability of Amino-Dextran Nanoparticles

**Figure 3:**
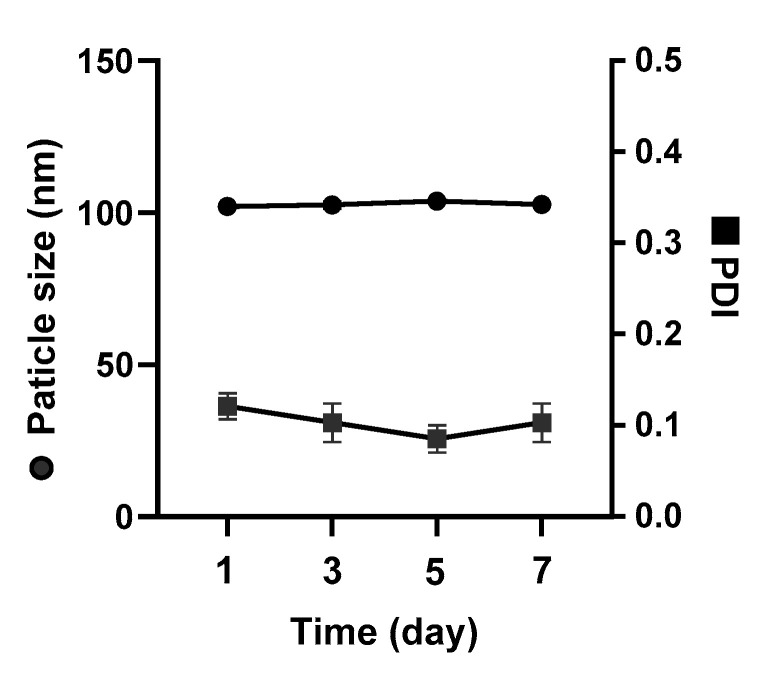
Colloidal stability of the amino-dextran nanoparticle suspension in water over a period of 7 days. Data are presented as the mean ± S.D. (*n* = 3).

**Figure 4:**
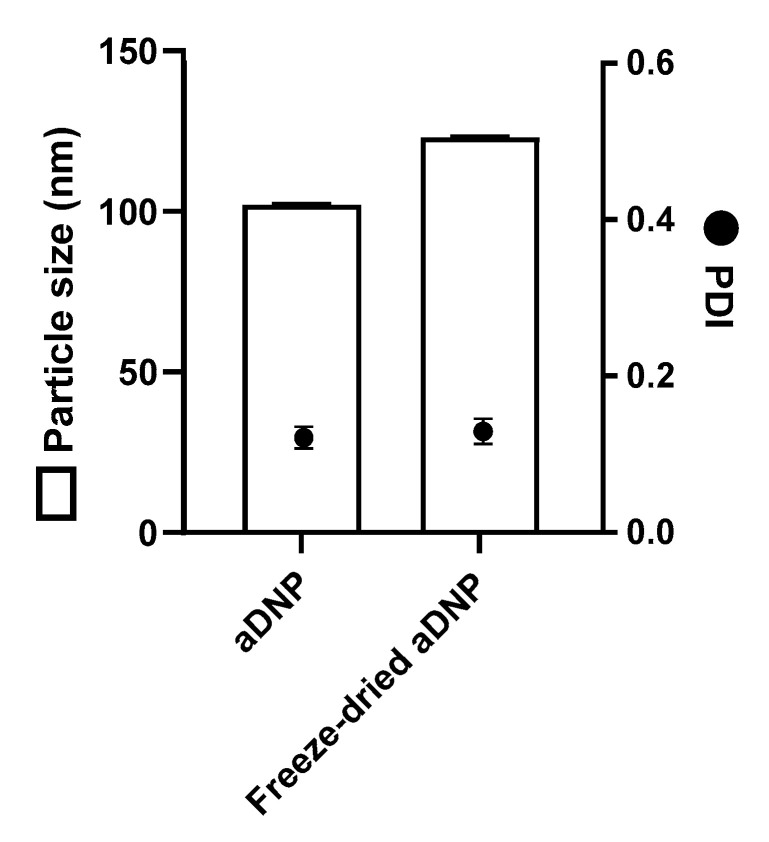
Freeze-drying of amino-dextran nanoparticle. Particle size and polydispersity index (PDI) of amino-dextran nanoparticles before and after freeze-drying. Data are presented as the mean ± S.D. (*n* = 3).

A stable nanoparticle formulation is a prerequisite for a safe and effective particulate delivery system, as the nanoparticle stability markedly affects cellular uptake, cytotoxicity, pharmacokinetics and biodistribution [23]. Short-term colloidal stability of nanoparticles in an aqueous solvent is important for downstream processing and administration. To investigate the short-term colloidal stability, aDNP (1 mg/mL dry weight equivalent) was prepared in water and the particle size was measured over 7 days at 4 °C. Under these conditions, Figure 3 shows that the particle size and the PDI did not change significantly over the 7-day period. For long-term storage, freeze-drying is a well-established technique to prolong the stability of the nanoparticles in the dry form. Often a cryoprotectant such as sucrose, trehalose, or dextran is required in the freeze-dried formulation to maintain nanoparticle stability [24]. Since dextran is used as a cryoprotectant, the freeze-drying of aDNP was performed without additional cryoprotectant. Freezing-drying of the aDNP suspension resulted in a cake that rapidly reconstituted upon addition of water. Figure 4 shows the particle size and the PDI of aDNP before and after freeze-drying. The freeze-drying process resulted in an increase in particle size from 100 to 120 nm, while did not have impact on the PDI.

### 3.3. CpG Loading onto Amino-Dextran Nanoparticles

**Figure 5:**
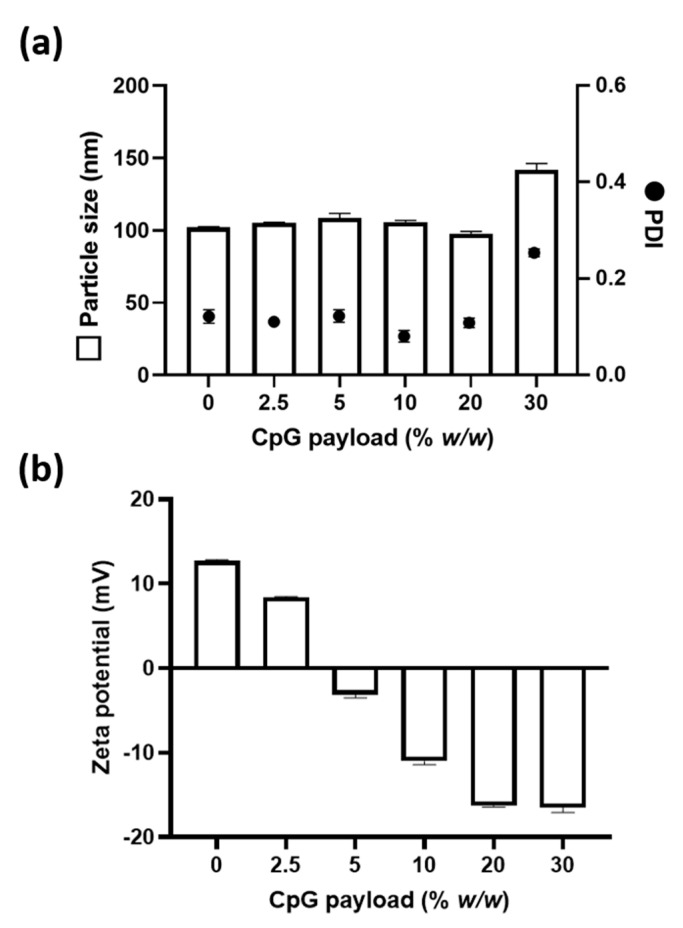
Effect of CpG payload (% *w/w*) on (**a**) particle size and PDI, and (**b**) zeta potential of the amino-dextran nanoparticle following CpG adsorption. Data are presented as the mean ± S.D. (*n* = 3).

The amine functionality of aDNP offers the possibility of electrostatic adsorption of anionic molecules onto the positively-charged particle surface. To demonstrate this capability, anionic CpG oligonucleotide was investigated as it has been recently shown to be a safe and effective vaccine adjuvant in humans [17]. aDNP was prepared from the amino-dextran that was functionalized with 50 amino groups per polymer, the particle size was 100 nm, PDI of 0.12, and a net positive charge of +12.5 mV. The CpG was adsorbed onto aDNP by adding various amounts of CpG to the aDNP suspended in water. After 4 h of incubation, the nanoparticle suspension was measured by DLS for particle size and zeta potential. Figure 5a shows that at a CpG payload of 2.5, 5, 10 or 20% (*w*/*w*), the particle size and PDI did not change significantly. However, at a 30% (*w*/*w*) CpG payload, both the particle size and PDI increased, and a second population of larger particles was observed in the size distribution histogram (Supplementary Figure S2).

A change in the zeta potential of a particle is an indicator for surface binding of molecules. To monitor the binding of CpG onto aDNP, the zeta potential was measured at the different CpG payloads (Figure 5b). The unloaded aDNP was cationic with a zeta potential of +12.5 mV, with addition of CpG at a payload of 2.5% (*w*/*w*) the cationic charge was reduced to +8.5 mV. At higher CpG payloads, the overall surface charge of aDNP became negative. From 5–20% (*w*/*w*) CpG payload, the zeta potential of the aDNP increased from −3.0 to −16.5 mV. At 30% (*w*/*w*) CpG payload, the zeta potential did not change significantly from the 20% (*w*/*w*) CpG payload, indicating saturation of CpG binding onto aDNP. These results showed that the addition of CpG to aDNP resulted in changes in the zeta potential of nanoparticle, suggesting CpG adsorption to aDNP.

**Figure 6:**
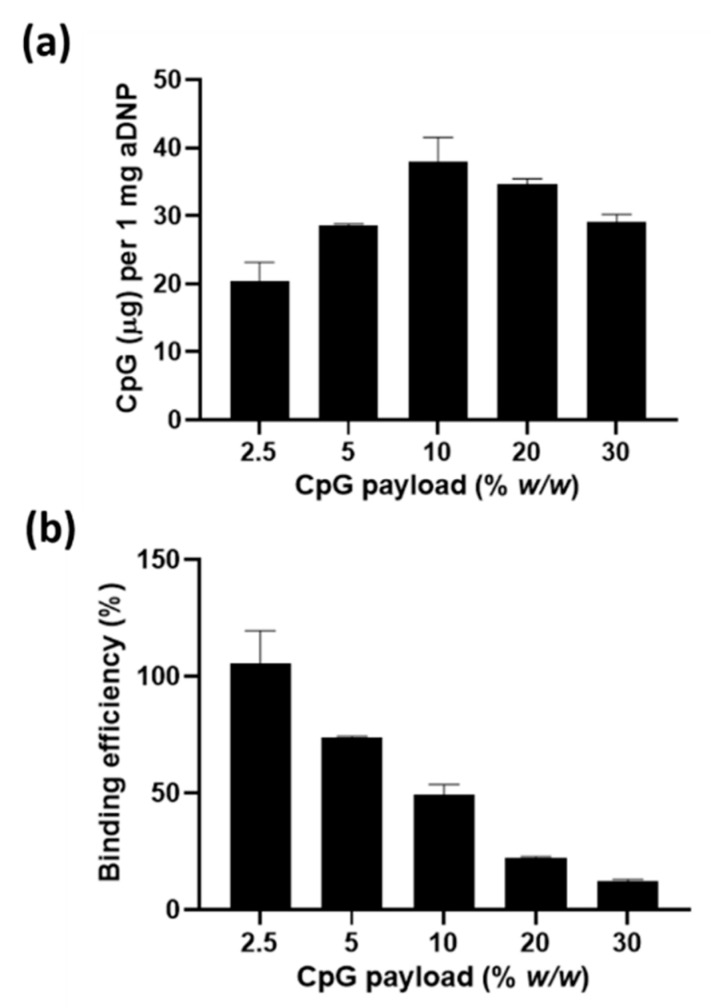
(**a**) Amount of CpG (µg) bound onto 1 mg of amino-dextran nanoparticles (dry weight equivalent) (**b**) CpG binding efficiency after loading different payloads of CpG on amino-dextran nanoparticle. Data are presented as the mean ± S.D. (*n* = 3).

To quantify the amount of CpG loaded onto aDNP, the unbound CpG was removed by centrifugal filtration (MWCO 10 kDa) and the nanoparticle suspension was assayed for CpG by the Quant-iT^®^ OliGreen^®^ ssDNA assay kit. This quantification assay uses a fluorescent nucleic acid stain that binds directly to oligonucleotides. The amount of CpG bound per mg of nanoparticle and the calculated binding efficiency is shown in Figure 6. At the lowest CpG payload of 2.5% (*w*/*w*), 20 µg of CpG was bound per mg of aDNP. At higher CpG payloads of 5 or 10% (*w*/*w*), the amount of CpG bound were 28 and 38 µg per mg aDNP, respectively. The amount of CpG bound to aDNP decreased at the payloads of 20 and 30% (*w*/*w*); however, this decrease was not significant.

A second CpG loading method was investigated, which involves the conjugation of CpG to the aDNP surface. CpG can be chemically synthesized with functional groups at either the 5′ or 3′ end to facilitate chemical conjugation [25]. In this study, the 3′-end of CpG modified with an amino group was conjugated to aDNP using the bis-aryl hydrazone linking strategy. The two-step conjugation process is shown in Supplementary Scheme S1. Using the same aDNP formulation as for the CpG adsorption studies, the amino group on the surface of the aDNP was reacted with the HyNic linker to introduce a hydrazine group (HyNic-aDNP). The amino-modified CpG was reacted with the 4FB linker to incorporate an aldehyde group on the 3′-end of the CpG molecule (4FB-CpG). In both reactions, the unreacted linker was removed by centrifugal filtration (MWCO 3kDa). CpG was then conjugated to aDNP by mixing 4FB-CpG with HyNic-aDNP to yield CpG-conjugated aDNP. This conjugation reaction was monitored by the change in the absorbance at 354 nm, which is the maximum absorbance wavelength of the formed bis-arylhydrazone linkage [26]. The formation of the stable bis-aryl hydrazone linkage between aDNP and CpG was confirmed by an increase in absorbance at 354 nm. The unreacted CpG was removed by centrifugal filtration (MWCO 10 kDa), and DLS analysis of the CpG-conjugated aDNP gave a particle size of 103 ± 0.1 nm, PDI of 0.145 ± 0.012 and zeta potential of CpG-conjugated aDNP was −12.5 ± 1.2 mV. The conjugation of CpG to aDNP resulted in nanoparticle with a similar particle size and PDI to the unmodified aDNP, while the zeta potential changed from +12.5 to −12.5 mV. This change in zeta potential indicated the immobilization of the negatively-charged CpG to the aDNP. The amount of CpG conjugated to aDNP was quantified using the Quant-iT^®^ OliGreen^®^ ssDNA assay kit, being 47.6 ± 0.7 µg CpG per 1 mg of aDNP.

### 3.4. Cytotoxicity and Hemocompatibility

**Figure 7:**
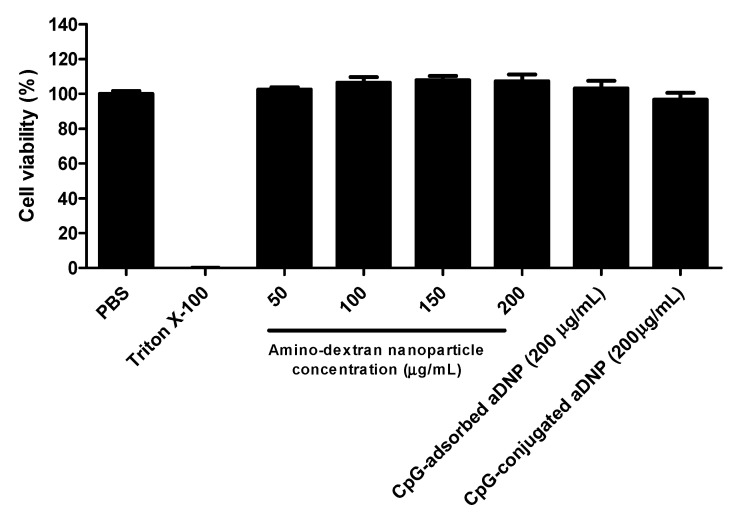
Cytotoxicity of unloaded aDNP and CpG-loaded aDNP on bone-marrow-derived dendritic cells after 48 h incubation. Data are presented as the mean ± S.D. (*n* = 3).

Dendritic cells are regarded as the most effective professional antigen-presenting cell for antigen processing and presentation to elicit an adaptive immune response and, therefore, are the main target for CpG formulations [14]. The cytotoxicity of the aDNP formulation with or without loaded CpG was evaluated on BMDCs using the MTS assay. To measure the cytotoxicity of the nanoparticles itself, unmodified aDNP was evaluated on BMDCs at 50, 100, 150, or 200 µg/mL, while CpG-loaded aDNPs was evaluated at 200 µg/mL. CpG was loaded onto aDNP either by the described adsorption or conjugation method. For the adsorption method, CpG was loaded with a payload of 10% (*w*/*w*). For the cytotoxicity assay, PBS and Triton-X100 were used as the negative and positive control samples, respectively. As shown in Figure 7, all aDNP formulations with or without CpG loading had the cell viability of greater than 95%.

Cationic nanoparticles have been reported to interact with the negatively-charged membrane of erythrocytes, which may result in thrombosis, embolization, and hemolysis in vivo [27]. For this reason, it is important to test the hemocompatibility of the positively-charged aDNP prior to in vivo studies. A percent hemolysis of less than 10% is considered as acceptable hemolytic activity for systemic application [28]. To evaluate the hemolytic activity of unloaded aDNP, the nanoparticles were suspended in PBS buffer (1 mg/mL). The deionized water and PBS buffer (pH 7.4) were used as positive and negative controls, respectively. For water, the percent hemolysis was 100% at 1 h, while for aDNP in PBS the percent hemolysis was 5.0 ± 1.6%.

### 3.5. In Vitro Cytokine Production

**Figure 8:**
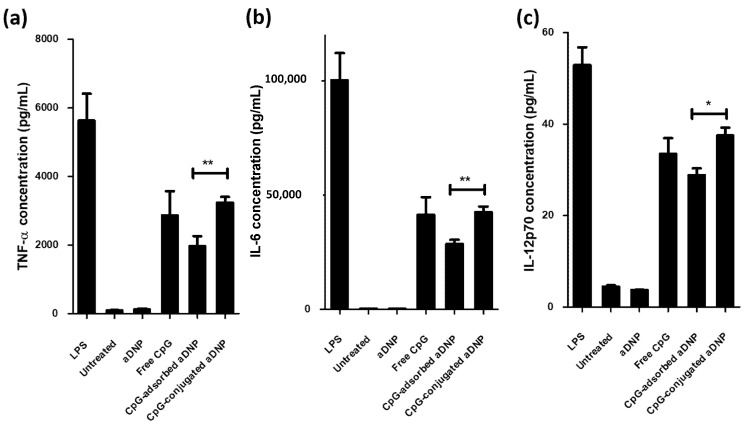
Production of cytokines, (**a**) TNF-α, (**b**) IL-6, and (**c**) IL-12p70, by bone marrow derived dendritic cells. The data are presented as mean ± standard error of the mean (S.E.M.) of three independent experiments. Statistical significance was determined with two-tailed Student’s *t* test. * *p* < 0.05; **, *p* < 0.01.

To assess the pro-inflammatory activity of the CpG formulations, the formulation was added to BMDCs and the levels of three pro-inflammatory cytokines secreted by the BMDCs were measured. TNF-α and IL-6 are key cytokines that are indicators for the maturation of dendritic cells [29,30], while IL-12p70 is important in generating T helper 1 (Th1) cells that subsequently support the proliferation of CD8^+^ cytotoxic T lymphocytes [14]. BMDCs were incubated with the CpG formulation at a fixed concentration of 0.4 µM. CpG was either in the free form or loaded onto the aDNP by the described adsorption or conjugation method. After 24 h incubation, the cytokines were measured using the bead-based LEGENDplex kit and analyzed by flow cytometry. Figure 8 shows that for all three cytokines, the cells treated with unloaded aDNP produced low cytokine levels similar to untreated cells. CpG-containing nanoparticle formulations stimulated cytokine production at significantly higher levels compared to untreated cells and unloaded aDNP, indicating that CpG loaded onto aDNP was able to activate BMDCs. For all cytokines tested, the cells treated with CpG-conjugated-aDNP formulation produced comparable levels of cytokines to those treated with free CpG, while the CpG-absorbed-aDNP showed a lower cytokine production compared to CpG and CpG-conjugated-aDNP.

## 4. Discussion and Conclusions

This work demonstrates that aDNPs can be prepared from amino-dextran polymer using the desolvation method. DLS and TEM measurements showed that the particles produced were in a nanoparticle size range with a spherical shape. The size and surface charge properties could be altered by varying the processing and formulation parameters (Figure 1 and Figure 2).

The particle size was shown to be dependent upon the anti-solvent used in the desolvation technique. This parameter was investigated with three commonly used anti-solvents for desolvation, including acetonitrile, acetone or ethanol. It was shown that the particle size decreased with increasing the water-miscibility of the solvent. This relationship between the particle size and the miscibility of solvent and anti-solvent has also been reported in previous research for the preparation of gelatin and PEG-PLGA nanoparticles [31,32]. It was hypothesized that smaller-sized nanoparticles produced with increasing water-miscibility could be attributed to better diffusion of water and dextran into the anti-solvent. To investigate the influence of water-to-antisolvent volume ratio on the particle size, the anti-solvent acetone was used. Increasing the ratio of water-to-acetone resulted in the formation of larger particles, and at the lowest water-to-acetone ratio of 1:3, a PDI of 0.42 was observed. A PDI of greater than 0.3 is considered as not sufficiently uniform particle size population for drug delivery purposes [33].

The level of amine functionality of the dextran could influence the crosslinking efficiency by glutaraldehyde and the surface charge of the nanoparticle. This work showed that the particle size and PDI decreased with increasing the level of amine functionality of the polymer. Zeta potential analysis of the particles showed that at the lowest amine functionality (10 amines) the surface charge was negative, while at higher functionality (25 and 50 amines) the particle surface was positive. The crosslinker content used in desolvation process to stabilize aDNP also influenced the particle size of aDNP. Increasing glutaraldehyde content has been shown to decrease the particle size, which was in agreement with the previous study on the desolvation of gelatin nanoparticles [34]. Taken together, these results provide the possibility of tuning the particle size and zeta potential of aDNP by varying formulation and production parameters to suit particular needs for drug delivery. Future experiments will investigate other parameters such as polymer concentration, stirring rate, or pH to modulate the particle size and zeta potential of aDNP.

Preliminary short-term colloidal stability for aDNP suspended in water was observed over 7 days at 4 °C (Figure 3). The particle size and PDI of aDNP were found to be unchanged over this period; therefore, aDNP are suitable for further downstream processes and manufacture. This work also showed that aDNP could be freeze-dried without additional cryoprotectants (Figure 4). This finding was expected, as dextran is a common cryoprotectant. Freeze-dried nanoparticle preparation without the need of cryoprotectant addition offers simplicity in formulation and product.

aDNP offers a versatile carrier by attaching active molecules via either electrostatic adsorption or covalent conjugation. In this study, CpG was investigated as it is anionic molecule that can be chemically modified for conjugation. The adsorption of CpG to the cationic aDNP was demonstrated by a change in the zeta potential with varying amounts of CpG payload (Figure 5 and Figure 6). The zeta potential ranged from +13 to −16 mV depending on the CpG payload added to aDNP. Based on the zeta potential, saturation of CpG adsorption was achieved at 20% (*w*/*w*) payload. At 30% (*w*/*w*) payload, the zeta potential remained unchanged; however, the particles size and PDI increased compared to 20% (*w*/*w*) CpG payload, indicating the particle instability.

The conjugation of CpG to aDNP was demonstrated using a bis-aryl hydrazone linking strategy, which has been shown to maintain the bioactivity of CpG in vitro and in vivo [35]. The successful conjugation of CpG to aDNP was observed by the change in the zeta potential of aDNP from being a cationic to anionic particle. Conjugation of CpG did not change the particle size (100 nm) and PDI. It was notable that the particle size, surface charge and CpG loading capacity of the aDNPs were similar for either loading method.

The aDNP platform was shown to have promising safety, using the MTS cytotoxicity assay both unloaded and CpG loaded aDNPs showed no cytoxicity on BMDCs (Figure 7). Although glutaraldehyde is considered as a toxic reagent [36], it was used in this study as it is commonly used for stabilizing nanoparticles and they have been shown to be non-toxic both in vitro and in vivo [14]. Future work should, however, examine alternative crosslinking agents for a better toxicity profile to reduce future regulatory hurdles.

One limitation of cationic nanoparticles is their propensity to damage the negative-charged cellular membrane of erythrocytes. This property was investigated by incubating the aDNPs with erythrocytes and then assessing for hemolysis. The static hemolysis assay showed promising low hemolytic activity for the aDNPs, indicating they could be used for systemic delivery. If the cationic surface properties are found to cause any signs of hemolysis on in vivo testing, this could be mitigated by surface immobilisation of anionic molecules or shielding polymers [37]. For example, in this study, it was shown that by loading CpG onto aDNP the zeta potential of nanoparticle became negative.

The ability of CpG-loaded aDNP to activate dendritic cells was evaluated on BMDCs by measuring the expression of the three pro-inflammatory cytokines, TNF-α, IL-6, and IL-12p70 (Figure 8). As expected, BMDCs that were exposed to lipopolysaccharide (LPS), a well-known immunostimulatory agent, showed the highest production for all three cytokines. The unloaded aDNP did not stimulate the immune cells confirming the carrier was not immunogenic in this assay, as the levels of cytokine production for all three markers were the same as buffer only. Free CpG and CpG-loaded aDNPs all resulted in elevated cytokine production and exhibited a similar profile for each cytokine (Figure 8a–c). Compared to CpG-adsorbed-aDNP, free CpG and CpG-conjugated-aDNP both demonstrated similar higher production for all three cytokines. This comparison indicates that the immobilization method of the CpG to the surface of the aDNP is important for immune stimulation. The lower immune stimulatory activity observed for the CpG-adsorbed-aDNP may be due to desorption of CpG from the nanocarrier prior to reaching the target TLR-9 receptor located in the endosome [35]. The desorption would subsequently reduce the CpG payload of aDNP and decrease the uptake of nanoparticles mediated by DEC-205, a surface receptor for CpG [38]. Previous studies investigating the immune response of CpG adsorbed on different cationic nanoparticles have shown both an enhancement and a reduction of immune-stimulation in vitro [14,39,40], while conjugation of CpG to nanoparticles has demonstrated enhanced in vitro immunostimulatory activity compared to free CpG [15,41]. To understand the observed in vitro differences between loading strategies, future studies will investigate the affinity of CpG to aDNP under various conditions and will also assess the in vitro uptake of the nanoparticles by immune cells. To determine if there is an in vitro-in-vivo correlation for loading strategy on immune stimulation, a comparative in vivo study should be undertaken. This immune stimulatory activity could be evaluated in a cancer animal model, which may also investigate the immunostimulatory effect enhancement of adding of specific peptide antigen to the nanocarrier. Similarly, Zhang et al. has shown an enhancement in the in vivo immune response to the model antigen ovalbumin when co-administered with a CpG conjugated to a dextran polymer in a melanoma cancer model [4].

This study is the first report on preparing dextran-based nanoparticle from a hydrophilic amino-dextran using the desolvation method. aDNP platform demonstrated potential advantages as a drug delivery platform such as; tunable physical properties, low toxicity, versatility for surface loading of molecules for tuning of the particle properties. This nanoparticle platform was evaluated as potential vaccine carrier however could also be used for the delivery of other bioactive molecules.

## Supplementary Materials

The following are available online at https://www.mdpi.com/1999-4923/12/12/1150/s1, Figure S1: Chemical structures of non-functionalized dextran and amino-functionalized dextran, Figure S2: Effect of CpG payload (% *w*/*w*) on the particle size distribution of the amino-dextran nanoparticle following CpG adsorption, Scheme S1: Schematic representation of the bis-arylhydrazone conjugation strategy to prepare the CpG-conjugated amino-dextran nanoparticle.
