# Supplementary material for: Nanoparticle System Based on Amino-Dextran as a Drug Delivery Vehicle: Immune-Stimulatory CpG-Oligonucleotide Loading and Delivery
Source: Pharmaceutics. 2020 Nov 27;12(12):1150. doi: 10.3390/pharmaceutics12121150 (PMC7760314; doi:10.3390/pharmaceutics12121150)
Supplement: Supplementary file 1 [file pharmaceutics-12-01150-s001.pdf]

pharmaceutics

Supplementary Materials: Nanoparticle System Based on Amino-Dextran as a Drug Delivery Vehicle: Immune-Stimulatory CpG-Oligonucleotide Loading and Delivery

Hien V. Nguyen, Katrin Campbell, Gavin F. Painter, Sarah L. Young and Greg F. Walker

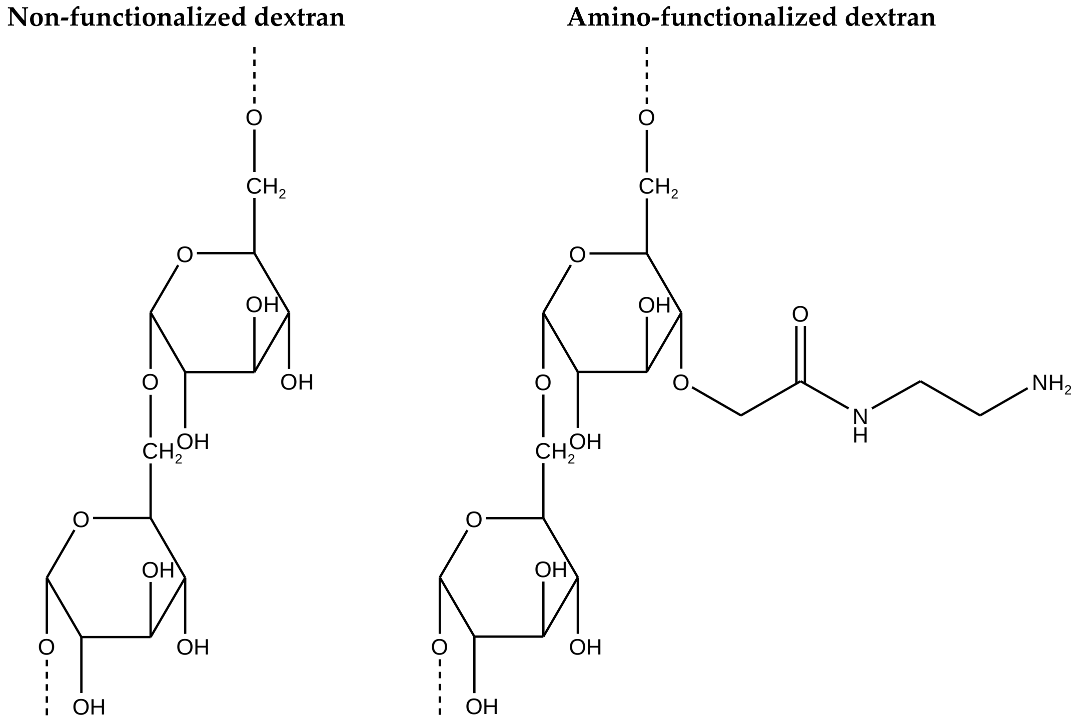

Figure S1. Chemical structures of non-functionalized dextran and amino-functionalized dextran.

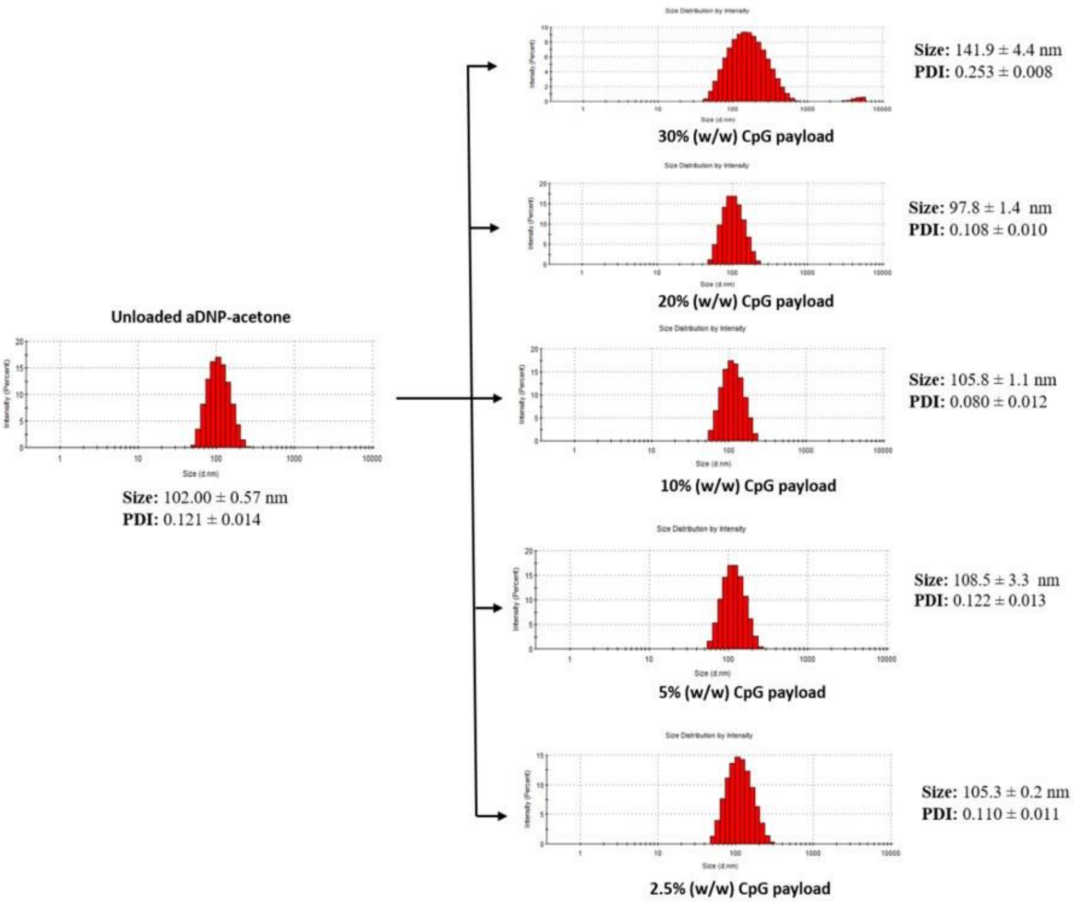

Figure S2. Effect of CpG payload (% *w*/*w*) on the particle size distribution of the amino-dextran nanoparticle following CpG adsorption.

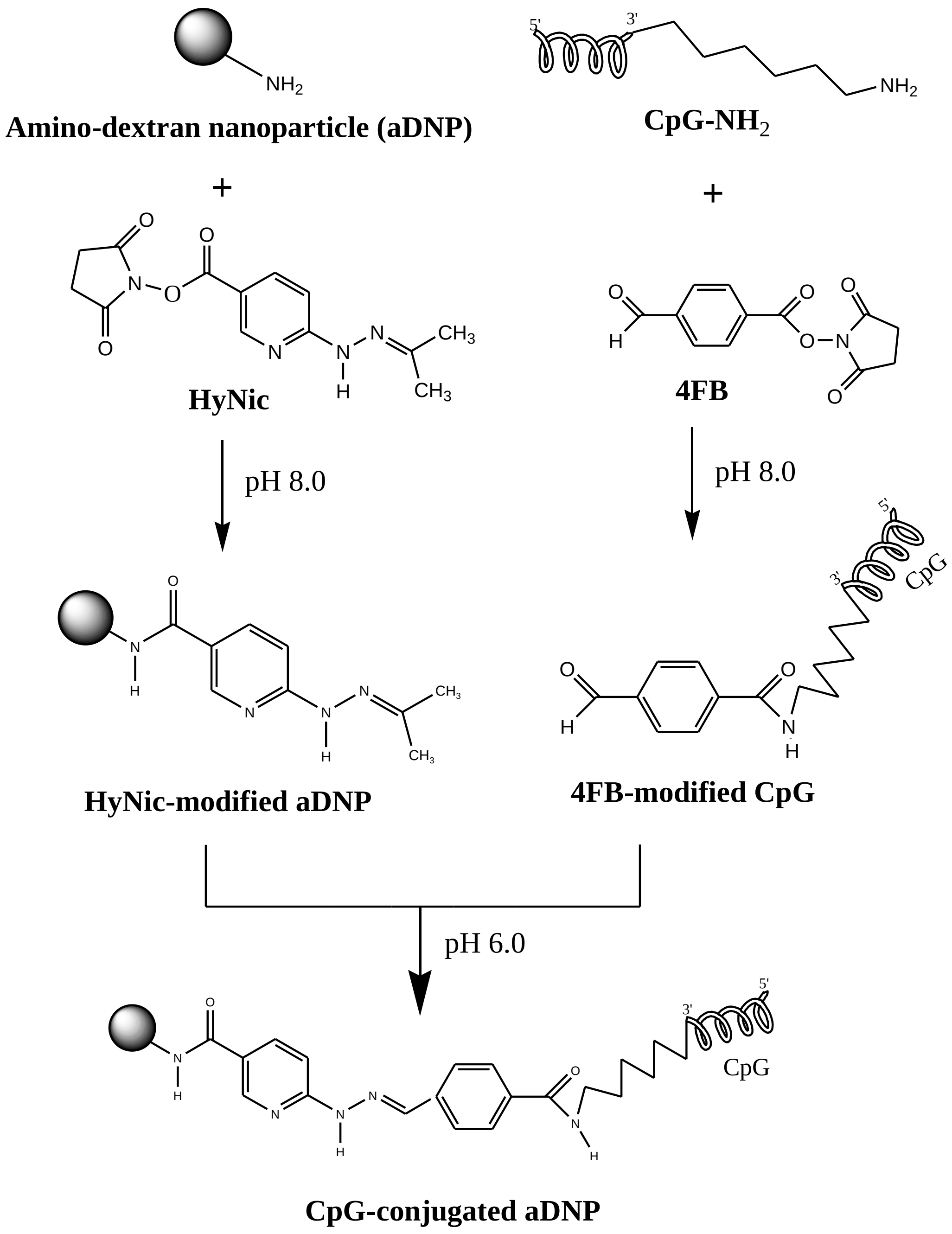

Scheme S1. Schematic representation of the bis-arylhydrazone conjugation strategy to prepare the CpG-conjugated amino-dextran nanoparticle.
